# Protection of the Furin Cleavage Site in Low-Toxicity Immunotoxins Based on *Pseudomonas* Exotoxin A

**DOI:** 10.3390/toxins8080217

**Published:** 2016-07-25

**Authors:** Gilad Kaplan, Fred Lee, Masanori Onda, Emily Kolyvas, Gaurav Bhardwaj, David Baker, Ira Pastan

**Affiliations:** 1 Laboratory of Molecular Biology, Center for Cancer Research, National Cancer Institute, National Institutes of Health, Bethesda, MD 20892, USA; gilad.kaplan@nih.gov (G.K.); fred.lee@nih.gov (F.L.); OndaM@mail.nih.gov (M.O.); emily.kolyvas@nih.gov (E.K.); 2 Department of Biochemistry, University of Washington, Seattle, WA 98195, USA; Gauravb@uw.edu (G.B.); dabaker@uw.edu (D.B.); 3 Institute for Protein Design, University of Washington, Seattle, WA 98195, USA; 4 Howard Hughes Medical Institute, University of Washington, Seattle, WA 98195, USA

**Keywords:** recombinant immunotoxin, mesothelin, *Pseudomonas* exotoxin A, disulfide bond

## Abstract

Recombinant immunotoxins (RITs) are fusions of an Fv-based targeting moiety and a toxin. *Pseudomonas* exotoxin A (PE) has been used to make several immunotoxins that have been evaluated in clinical trials. Immunogenicity of the bacterial toxin and off-target toxicity have limited the efficacy of these immunotoxins. To address these issues, we have previously made RITs in which the Fv is connected to domain III (PE24) by a furin cleavage site (FCS), thereby removing unneeded sequences of domain II. However, the PE24 containing RITs do not contain the naturally occurring disulfide bond around the furin cleavage sequence, because it was removed when domain II was deleted. This could potentially allow PE24 containing immunotoxins to be cleaved and inactivated before internalization by cell surface furin or other proteases in the blood stream or tumor microenvironment. Here, we describe five new RITs in which a disulfide bond is engineered to protect the FCS. The most active of these, SS1-Fab-DS3-PE24, shows a longer serum half-life than an RIT without the disulfide bond and has the same anti-tumor activity, despite being less cytotoxic in vitro. These results have significance for the production of de-immunized, low toxicity, PE24-based immunotoxins with a longer serum half-life.

## 1. Introduction

Recombinant immunotoxins (RITs) are fusions of a targeting moiety and a toxin [1]. While some immunotoxins use a natural ligand to target a specific receptor, others use an Fv or Fab fragment of a receptor-targeting antibody. *Pseudomonas aeruginosa* (PE)-based immunotoxins usually contain a 38 kDa fragment of the *Pseudomonas aeruginosa* exotoxin A (PE38), consisting of domains II and III of the native toxin; domain I is replaced by the Fv.

Once the Fv portion of the immunotoxin binds to its target receptor, the immunotoxin is internalized by endocytosis. In the endocytic compartment, the immunotoxin is cleaved by the pro-protein convertase (PC) furin, leading to the formation of two immunotoxin fragments; the Fv containing fragment and a second fragment consisting of most of domain II and the catalytic domain III. These two fragments are held together by a disulfide bond. The disulfide bond is reduced and the catalytic fragment is trafficked by late endosomes to the trans-golgi network (TGN), and there binds to the KDEL receptor, which traffics it to the endoplasmic reticulum (ER). From there, the toxin fragment is translocated into the cytosol. Once in the cytosol, domain III catalyzes the adenosine diphosphate ribosylation of a diphthamide modification on elongation factor 2 (eEF2), thereby inhibiting protein translation and ultimately causing apoptosis [2,3,4].

Immunotoxins have been investigated in several clinical trials for treating cancer (reviewed in [2,3,4,5]). Some of the main challenges identified in these trials is non-specific animal toxicity and high immunogenicity producing anti-drug antibodies (ADA) that hamper immunotoxin efficacy [5]. Clinical trials with immuno-suppressed leukemia patients, whose immune status diminished ADA formation, showed higher immunotoxin efficacy [5,6].

**Figure 1:**
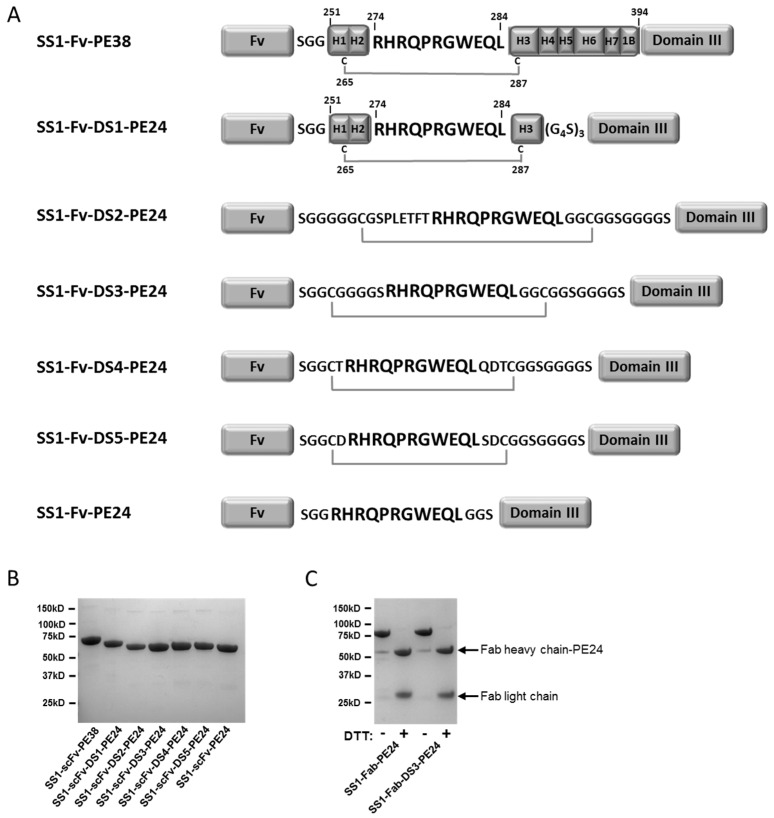
Design and production of DS-PE24 constructs. (**A**) Sequences connecting the antibody Fv (Ab) and PE domain III. PE domain II contains seven helixes (H1–H7), domain IB and the furin cleavage site (FCS, “RHRQPRGWEQL”). Note the two naturally occurring cysteines constraining the FCS in this context. In SS1-Fv-PE24 toxin domain II has been replaced by the FCS flanked by short linkers. The modified linkers of the various DS-PE24 mutants are shown. Numbering refers to the mature full length *Pseudomonas* exotoxin A; (**B**) SDS-PAGE of the produced scFv immunotoxins; (**C**) SDS-PAGE of the produced Fab immunotoxins.

To try and prevent processing of PE38 toxins by antigen presenting cells, thereby reducing the immunogenicity of PE-based immunotoxins, a lysosomal protease resistant version of PE38 (designated “PE24”) was previously developed [7]. While PE38 consists of two domains (domains II and III) of the full length exotoxin A, the PE24 toxin consists of only the catalytic domain III connected to the targeting Fv by the furin cleavage site (FCS) normally found in domain II (see Figure 1). By removing domain II, processing by lysosomal enzymes is reduced, and immunogenic epitopes found within domain II are also removed. When combined with point mutations designed to disrupt B and T cell epitopes in the remaining domain III, a de-immunized PE24 molecule was produced, which was shown to be significantly less immunogenic in mouse models [8,9]. In addition to low immunogenicity, PE24 toxins show improved cytotoxic activity in multiple cell lines compared to PE38 toxins in vitro, and much lower non-specific toxicity in mice and rats, allowing higher doses to be given [10,11]. PE24 toxins therefore exhibit improved characteristics for clinical trials. However, in re-engineering of PE38 into PE24, the native disulfide bond around the FCS in domain II of PE38 was removed, leaving only a non-constrained FCS sequence between toxin domain III and the Fv targeting moiety.

Furin is a ubiquitous transmembrane serine endoprotease [12]. As a member of the PC family, furin cleaves multiple pro-proteins along the secretory pathway [12]. Transmembrane furin cycles between the TGN, the cell surface and endosomal compartments [12,13]. Production of a soluble, secreted form of furin has also been described in epithelial cells in vitro by posttranslational processing. Furin cleavage is necessary for activation of several toxins, including pseudomonas, diphtheria and anthrax toxins [12]. While PE-based toxins are activated by furin cleavage after internalization in the early endosome, anthrax toxin is activated by furin cleavage at the cell surface, showing that furin is present and active at the plasma membrane [14]. PE38 immunotoxins contain a naturally occurring disulfide bond around the FCS found in domain II. If these immunotoxins are cleaved by cell surface furin, the disulfide bond will prevent disassociation of the toxin and Fv until it is reduced within the cell following immunotoxin internalization. In contrast, the unprotected FCS found in PE24 based immunotoxins, lacking a protective disulfide bond, could be cleaved by furin in the tumor extra-cellular environment or at the cell surface before internalization, thereby deactivating PE24-based immunotoxins.

In this paper, we describe the design and preparation of RITs in which the furin cleavage site is stabilized by addition of different peptide bridges that form a disulfide bond across the furin site so that the two portions of the immunotoxin remain together until the disulfide bond is reduced. We have then characterized the properties of the most promising of these RITs.

## 2. Results

### 2.1. Design and Production of the DS-PE24 Immunotoxins

To bridge the furin cleavage site (FCS, “RHRQPRGWEQL”) with a disulfide bond, cysteine containing flanking peptides were designed and introduced into the anti-mesothelin SS1-PE24 immunotoxin (Figure 1A). The first DS-PE24 construct is SS1-Fv-DS1-PE24. It retains helixes 1–3 of domain II (residues 251–301), which include the naturally occurring disulfide bond and the FCS, surrounded by Gly-Ser linkers. As removal of helixes 4–7 uncovers the hydrophobic core of domain II, we anticipated solubility problems with this construct. Therefore, four hydrophobic residues in the uncovered core were mutated into polar residues in order to increase solubility (L256Q, L259Q, V290T and L293Q).

SS1-Fv-DS2-PE24 contains helix 2 and the FCS, surrounded by long Gly-Ser linkers with two cysteines located the same number of residues away from the furin site as in the native domain II. SS1-Fv-DS3-PE24 is the simplest of the DS-PE24 constructs. The 11 amino acid FCS is bridged by two cysteines, six glycines and one serine.

The final two constructs, SS1-Fv-DS4-PE24 and SS1-Fv-DS5-PE24, contain sequences surrounding the FCS that were designed computationally using Rosetta Protein Design methods [14,15] to constrain the FCS in the native conformation found in domain II.

Because RITs combining a scFv and PE24 have a low molecular weight of around 50 kDa, and are subject to renal filtration resulting in a short in vivo half-life, the scFv targeting moiety was replaced with a Fab to increase size, reduce filtration by the kidney and increase half-life. Therefore, the parental SS1-PE24 immunotoxin and the SS1-DS3-PE24 construct were also produced as SS1-Fab immunotoxins (SS1-Fab-PE24 and SS1-Fab-DS3-PE24). All of the RITs were produced by standard protein refolding from *Escherichia coli* (*E. coli*) inclusion bodies (see Materials and methods) and were obtained as highly purified monomeric proteins of the correct molecular weight (Figure 1B,C). Starting with 100 mg of total protein, all the RITs could be produced in large amounts by our standard refolding procedure (Table 1).

### 2.2. The Disulfide Bond around the Furin Cleavage Site Is Formed

To show that a disulfide bond had formed around the FCS in our DS-PE24 immunotoxins, we treated the DS-PE24 proteins with soluble recombinant furin. We expected that even after furin cleavage, the DS-PE24 immunotoxins would still migrate as a single band on a non-reducing gel, because the two fragments would still be connected by the disulfide bond. We also expected that they would migrate as two bands in a reducing gel, because the disulfide bond would then be reduced.

**Figure 2:**
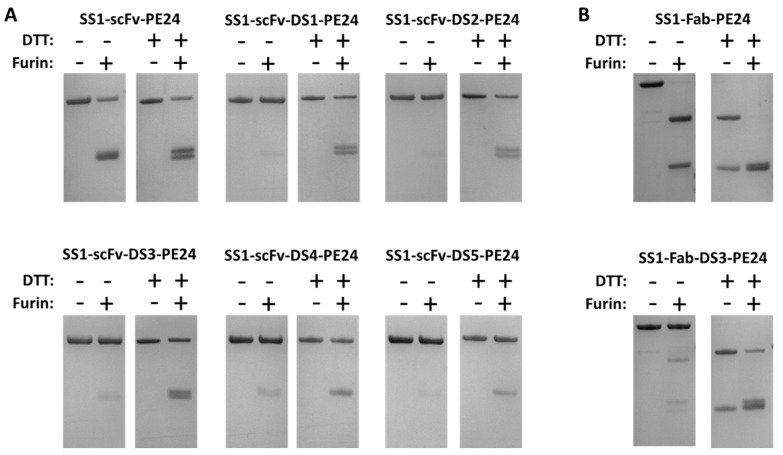
Furin cleavage verifies the presence of the disulfide bond around the furin cleavage site. (**A**) ScFv and (**B**) Fab constructs were cleaved with recombinant furin and analyzed by SDS-PAGE under reducing and non-reducing conditions. A disulfide bond around the furin cleavage site should keep the antibody Fv and toxin parts of the immunotoxin associated until they are reduced. All constructs showed enhanced disassociation under reducing conditions, indicating the formation of the FCS-protective disulfide bond. Susceptibility to cleavage by furin varied between the DS-PE24 constructs, with the SS1-scFv-DS5-PE24 construct showing the least amount of furin cleavage.

As shown in Figure 2, the parental SS1-scFv-PE24, which does not contain a disulfide bond between the toxin and the Fv, shows a lower molecular weight band after furin cleavage under non-reducing conditions, indicating separation of immunotoxin into an Fv and a toxin fragment with similar molecular weights (25.5 and 26.7 kDa, respectively). These two fragments are better resolved, when the proteins are run under reducing conditions, presumably because the disulfide bonds in the scFv are reduced changing the shape of the protein. Using soluble furin, we were unable to reach 100% immunotoxin cleavage, even under optimized conditions.

In contrast, SS1-scFv-DS1-PE24 and all the other DS-PE24 proteins migrated as a single band on a non-reducing gel when treated with furin. Only when analyzed on a non-reducing gel were low molecular weight bands after furin treatment observed, which correspond to the scFv and PE24. In all cases, some un-cleaved DS-PE24 protein remained, because the soluble furin could not digest all the added protein. The extent of cleavage varied among the five proteins. ScFv immunotoxins containing the DS1, DS2, or DS3 sequences were cleaved more completely than SS1-scFv-DS4-PE24 or SS1-scFv-DS5-PE24.

We also examined the cleavage of the two Fab immunotoxins with furin. As shown in Figure 2B, SS1-Fab-PE24 was almost completely cleaved by furin as indicated by the disappearance of the high molecular weight band in the gel. The products represent the Fab and PE24. In contrast, furin treatment of SS1-Fab-DS3-PE24 had very little effect on the high molecular weight band when the protein was analyzed on a non-reducing gel, but the protein dissociated into lower molecular bands on a reducing gel after furin treatment. Under reducing conditions, the V_L_-κ chain is also visible as part of the lower double band.

Overall, the formation of a FCS protective disulfide bond was observed in all of the DS-PE24 mutants to some degree. We could not fully quantify the percentage of molecules containing the disulfide bond due to lack of full cleavage by furin.

### 2.3. Cytotoxicity Assays

The cytotoxic activity of the different DS-PE24 immunotoxins was compared in a WST-8 cell growth inhibition assay on four different cell lines; the pancreatic cancer KLM-1 cell line, the mesothelioma HAY cell line, the gastric cancer MKN45 cell line and the lung cancer L55 cell line. All of these cancers express mesothelin, the target of the SS1 targeting moiety found in the tested immunotoxins. KLM-1 cells express high levels of mesothelin (60 × 10^3^ sites/cell) [15], as do HAY cells (unquantified) [16] and MKN-45 cells (9.9 × 10^3^ sites/cell) [17]. On the other hand, L-55 cells express much lower amounts of mesothelin (unquantified) [18,19].

**Figure 3:**
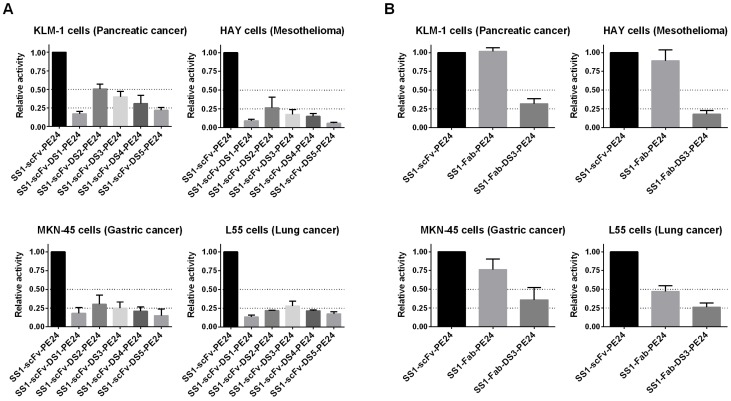
Addition of the disulfide bond around the furin cleavage site leads to a reduction in cytotoxic activity. The IC_50_ (pM) of the parental and DS-PE24 constructs on four mesothelin expressing cancer cell lines representing four different cancers was established using a WST-8 growth inhibition assay. ScFv immunotoxins are shown in (**A**) and Fab immunotoxins in (**B**). The tested cell lines were the pancreatic cancer KLM-1, mesothelioma HAY, gastric cancer MKN-45 and lung cancer L55. The results from 3 to 4 experiments were plotted as relative activity with respect to the parental SS1-scFv-PE24 immunotoxin. Actual IC_50_ values (pM) are shown in Table 2. The DS-PE24 constructs were overall less active than the parental immunotoxin, with the most active mutants (DS2 and DS3) showing a 2–6-fold activity reduction, depending on the cell line. Error bars represent SEM.

As shown in Figure 3A and Table 2, the most active DS-PE24 constructs were SS1-scFv-DS2-PE24 and SS1-scFv-DS3-PE24. However, they were about 2–6-fold less active than the parental SS1-scFv-PE24, depending on the cell line studied. SS1-scFv-DS1-PE24, which contains a large portion of domain II, exhibited much lower cytotoxic activity, with a reduction of 6-11 fold in cytotoxic activity. Of the two computationally designed linkers, SS1-scFv-DS4-PE24 exhibited cytotoxic activity that was only slightly lower than SS1-scFv-DS2-PE24 and SS1-scFv-DS3-PE24, while SS1-scFv-DS5-PE24 showed the lowest activity of the constructs with a 5–18-fold reduction.

Figure 3B and Table 2 show data with the Fab containing immunotoxins. We found that SS1-Fab-PE24 and SS1-Fab-DS3-PE24 showed cytotoxic activities similar to the scFv immunotoxins. Since immunogenicity is a problem with PE immunotoxins, using a linker peptide with the least possible immunogenic sequences would be advantageous. The DS3 linker combines maximal cytotoxic activity with a linker consisting of glycines and serines that are not usually immunogenic. We therefore focused on DS-PE24 immunotoxins containing the DS3 linker in subsequent assays.

### 2.4. Stability Studies

The DS-PE24 immunotoxins were constructed to protect the FCS from extra-cellular cleavage by furin or other proteases in the circulation. To determine if the addition of the disulfide bond affects stability, both the parental PE24 and DS3-PE24 immunotoxins were tested in a serum stability assay (Table 3). Both scFv immunotoxins (SS1-scFv-PE24 and SS1-scFv-DS3-PE24) and Fab immunotoxins (SS1-Fab-PE24 and SS1-Fab-DS3-PE24) were incubated in 95% human serum at 37 °C for 1, 6 and 24 h, and then tested for cytotoxic activity on the human pancreatic cell line KLM1. All molecules except SS1-Fab-DS3-PE24 showed no significant changes up to 6 h and a significant 1.6–2-fold decrease after 24 h (uncorrected Fisher’s LSD, *p* < 0.05). SS1-Fab-DS3-PE24 showed no significant changes over the 24 h period. While the two DS-PE24 constructs, SS1-scFv-DS3-PE24 and SS1-Fab-DS3-PE24, seemed slightly more stable than the PE24 immunotoxins; these differences were not statistically significant (uncorrected Fisher’s LSD). We conclude that the addition of the disulfide bond around the FCS does not affect overall immunotoxin stability.

### 2.5. Half-Life Studies in Mice

**Figure 4:**
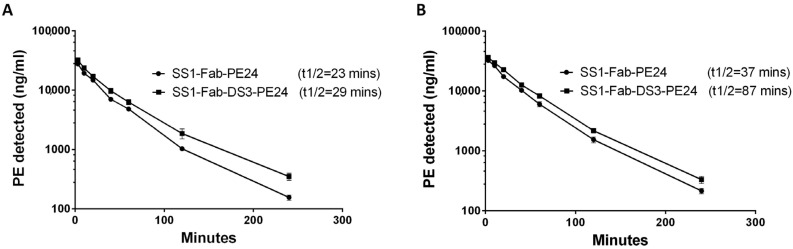
The added disulfide bond increases the serum half-life of DS-PE24 immunotoxins. Mice (*n* = 3) were injected with either the wild-type SS1-Fab-PE24 or SS1-Fab-DS3-PE24 immunotoxins and PE serum levels were monitored over time (3, 10, 20, 40, 60, 120, and 240 min) using sandwich ELISA. The clearance of the two immunotoxins was analyzed using a “two-phase decay” model. In both experiments (**A**,**B**), SS1-Fab-DS3-PE24 showed a significant increase in half-life, from 22.2 to 29.8 min (34%) in the first experiment (**A**) and from 37.6 to 87.9 min (133%) in the second experiment (**B**). The serum PE levels are detailed in Table 4. Error bars represent SEM.

The Fab constructs (SS1-Fab-PE24 and SS1-Fab-DS3-PE24) were chosen for mouse studies because they were very active in vitro and are expected to have a longer half-life and more activity in mice. To measure the half-life, mice were injected with 30 μg of the SS1-Fab-PE24 or SS1-Fab-DS3-PE24 (three mice in each group). This dose is similar to the dose used to treat tumors in mice. Blood samples were obtained at different times, and the serum levels of immunotoxin were detected using ELISA (Figure 4 and Table 4). The half-life of the two immunotoxins was compared using a “two-phase decay” model that models the clearance of the drug into two phases, an initial fast phase and a slower second phase.

As shown in Figure 4 and Table 5, addition of the DS linker increased the slow phase half-life of SS1-Fab-DS3-PE24 from 23 to 29.8 min (a 29% increase) in the first experiment and from 37.6 to 87.9 min (a 133% increase) in the second experiment. In both experiments, the addition of the disulfide bond around the FCS caused a decrease in clearance in both the fast and slow clearance phases, as well as an increase in the area under the curve (AUC) (Table 5). The reason for the difference in half-lives between the two mouse experiments is probably due to the different amounts of blood drawn for the blood level ELISAs (40 µL vs. 10 µL), because the larger volumes removed substantial amounts of immunotoxin from the blood stream.

### 2.6. Anti-Tumor Experiments

**Figure 5:**
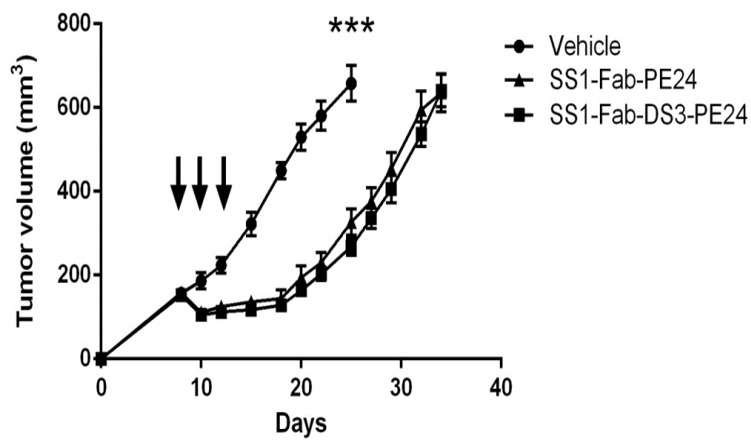
The SS1-Fab-DS3-PE24 immunotoxin is as active as the parental SS1-Fab-PE24 immunotoxin in vivo. Athymic nude mice were inoculated subcutaneously with cells from the human pancreatic cell line KLM1. Once the tumors reached a volume of 150 mm^3^, they were treated with either vehicle (*n* = 7), the parental SS1-Fab-PE24 (*n* = 10) or SS1-Fab-DS3-PE24 (*n* = 10) on days 8, 10 and 12 (indicated by arrows). Tumors were measured every 2–3 d until volumes reached 600 mm^3^. At day 25, the vehicle group had significantly larger tumors (Mann-Whitney, *p* < 0.0001) than the treated groups, but no significant differences in anti-tumor activity were seen between the parental SS1-Fab-PE24 and the SS1-Fab-DS3-PE24 treated groups throughout the experiment. Mice weights remained stable throughout the experiment. Error bars represent SEM.

The anti-tumor activity of SS1-Fab-DS3-PE24 was compared with the parental SS1-Fab-PE24 immunotoxin with respect to the growth of pancreatic cancer KLM-1 tumors in mice. Mice were implanted with 5 million KLM-1 tumor cells in the right flank on day 0 and treatment began on day 8 when the tumors reached about 150 mm^3^ in size. Mice were treated every other day (×3) with either 50 μg of either RIT or saline and tumor size monitored over several weeks. As shown in Figure 5, both immunotoxins show a 9-day tumor growth delay (from 25 days to 34 days) to reach a tumor volume of 600 mm^3^, but showed no significant difference from each other. None of the treated mice showed significant weight loss, showing the given doses to be well tolerated. Noticeably, the SS1-Fab-DS3-PE24 immunotoxin, while being less active in vitro, shows wild-type anti-tumor activity in vivo.

## 3. Discussion

### 3.1. DS-PE24 Immunotoxins Are Producible, Stable and Cytotoxic

In the study presented here, a disulfide bond was engineered around the non-constrained FCS connecting the targeting Fv and the PE24 toxin. We were able to successfully make several of these proteins by simply inserting a cys reside before and after the furin site, leaving a sufficient number of amino acids between the cys residues for a disulfide bond to form. During the immunotoxin re-folding step, which contains a redox reagent, the disulfide bonds formed in an efficient manner. The proteins we produced could be readily purified and were stable and cytotoxic to target cells, although their cytotoxicity in cell culture was reduced compared to the non-disulfide bonded parental immunotoxin. Despite this, the SS1-Fab-DS3-PE24 had similar in vivo activity as the parental SS1-Fab-PE24 immunotoxin with respect to tumors from a pancreatic cancer cell line.

### 3.2. Furin Cleavage Correlates with Immunotoxin Activity

As discussed above, cleavage by furin is a necessary step for intoxication with SS1-PE immunotoxins. In our in vitro furin cleavage assay, the construct that showed the least cleavage by furin was SS1-scFv-DS5-PE24 (Figure 2). This construct, as well as SS1-scFv-DS4-PE24, contained bridging sequences computationally designed to constrain the FCS in its native conformation. Interestingly, this immunotoxin also showed the lowest cytotoxic activity in vitro (Figure 3). This points towards one possible explanation for the generally lower cytotoxic activities of the DS-PE24 immunotoxins, i.e., that our disulfide-constrained furin cleavage sequences undergo furin cleavage at lower efficiency, leading to lower cytotoxic activity. It may be possible to further optimize the FCS bridging sequences by systematically optimizing the number of amino acids within the disulfide bridged loop (between the cysteines and the FCS) and between the disulfide bridged loop and the Fv and toxin, thereby optimizing furin cleavage efficacy and regaining the loss in cytotoxic activity.

### 3.3. Importance of Serum Half-Life of Protein Therapeutics

The serum half-life of a protein therapeutic is of great importance to its overall efficacy [20]. Many such therapeutics show increased effectiveness when beneficial concentrations are maintained for longer periods. The half-life of a protein therapeutic is determined by the sum of its physicochemical characteristics, which determines how susceptible the protein will be to the different clearance mechanisms [20,21]. Clearance mechanisms include hepatic elimination, renal filtration of molecules smaller than 50 kDa, receptor mediated endocytosis and proteolysis by peripheral blood cells. General strategies to increase serum half-life include increasing molecule size to over 50 kDa, fusion to an IgG Fc, PEGylation or conjugation to albumin binding motifs [20].

In this study, we focused on the effect of protecting the unconstrained FCS found in PE24 immunotoxins. Our results indicate that an unprotected FCS leads to a lower serum half-life (Figure 4). While the exact mechanism of clearance is unclear, the nature of the FCS suggests that furin, or another protease, cleaves the unprotected FCS in the blood stream, thereby separating the immunotoxin into two smaller Fv and toxin fragments that are rapidly cleared. Protecting the FCS using a bridging disulfide bond precludes this separation by physically bridging the two fragments until it is reduced after internalization, thus extending immunotoxin half-life. This increased stability could potentially be even more significant within the tumor microenvironment than in the bloodstream, for reasons discussed below.

### 3.4. Importance of Proteolytic Stability in the Tumor Microenvironment

The tumor microenvironment has been shown to play an important part in tumor progression, metastasis and resistance to therapeutic agents [22,23]. Resistance to protein therapeutics is thought to be a result of, among others, lack of access to the tumor due to poor vasculature, secretion of growth factors by the tumor-supporting matrix and upregulation of proteases. The immunotoxin activating endoprotease furin has been found to be upregulated in several cancers, including breast cancers [24], non-small-cell lung carcinomas [25] and some squamous cell carcinomas [26,27]. In addition, tumor hypoxia has been shown to cause furin to localize to the cell surface in fibrosarcoma cells [28]. Together, there is evidence to suggest that cell surface furin will be present in larger amounts within solid tumors. Why are we specifically concerned with extracellular furin cleavage within the tumor micro-environment, when the serum half-life does not seem to be affected much by this additional proteolytic clearance? Previous experiments published by our laboratory studied the in vivo uptake of immunotoxins into tumors from a human pancreatic cancer cell line implanted in athymic nude mice [29]. These experiments showed that maximal uptake of immunotoxin occurred after 4–6 h. This means that although the serum half-life in mice is usually 20–30 min [30], immunotoxins need to remain stable for at least ten times longer within the tumor microenvironment. The situation in human tumors should be similar, or even worse, as serum half-lives are longer and tumors grow to be larger. Therefore, protecting PE24 immunotoxins from extracellular disassociation due to cleavage should prolong their stability and as a result their efficacy.

### 3.5. Summary

In summary, removal of toxin domain II from PE38 to create the PE24 toxin is critical in order to reduce general non-specific toxicity and to remove highly immunogenic sequences. In addition, point mutations that disrupt T and B cell epitopes to further reduce immunogenicity have, to date, only been found for PE domain III. Thus, only PE24 immunotoxins, and not PE38 immunotoxins, can be converted into low immunogenicity toxins. We show that the unprotected linear FCS sequence in PE24 immunotoxins is susceptible to rapid clearance, and hypothesize that FCS proteolysis can occur at elevated levels within the solid-tumor microenvironment. Engineering a disulfide bond around the FCS represents a working strategy to extend the in vivo stability of PE24 immunotoxins.

## 4. Materials and Methods

### 4.1. Construction, Expression and Protein Purification

Double-stranded gene fragments (gBlocks, Integrated DNA Technologies, Coralville, IA, USA) corresponding to the desired sequences were ordered and ligated into our standard laboratory immunotoxin production vector as described previously [31] using the Gibson Assembly Master Mix (New England Biolabs, Ipswich, MA, USA) according to manufacturer’s instructions. All RITs were expressed and purified from *E. coli* inclusion bodies as previously described [31]. Briefly, inclusion bodies from *E. coli* were prepared and dissolved in GTE buffer (6 M guanidine-HCl, 100 mM Tris-HCl, 2 mM EDTA). The denatured proteins were then refolded in 100 mL Tris-HCl, 1 mM EDTA, 0.5 M arginine, 0.5 M NDSB-201, pH 10 for 32 h, and then dialyzed against 30 mM Tris-HCl, 0.1 M urea for 40 h. The refolded proteins were purified using ion-exchange and size exclusion chromatography.

### 4.2. Computational Design of DS-PE24 Linkers

Rosetta Protein Design methods [32,33] were used to design linkers that constrain the FCS in the native conformation found in domain II. For this, the furin cleavage site (FCS) was extended by appending 2–5 amino acids on each side and the backbone conformations of these appended residues were sampled in torsional space and analyzed for placement of disulfide bonds with ideal geometry. Around 20,000 different sequences were designed using Rosetta sequence design methods [33], and two of the lowest energy sequences were selected for experimental characterization (DS4 and DS5).

### 4.3. Cell Growth Inhibition Assay

The cytotoxic activity of the RITs was evaluated using the WST-8 cell counting kit (Dojindo Molecular Technologies, Rockville, MD, USA). Cells were incubated with varying concentrations of immunotoxin for 72 h, after which the WST-8 reagent was added. Ninety-six well plates were read at OD 450. Readings were normalized to the phosphate buffered saline (PBS) only positive control and the IC_50_ (concentration inhibiting growth by 50%) for each construct was calculated using a variable four-parameter slope, non-linear regression fit with Graphpad Prism vs. 6.01. One hundred percent cell killing was achieved using 2.3 μg/mL of staurosporine (Sigma, St. Louis, MO, USA) as a control. Each assay contained four replicates of each concentration and the assays were repeated three to four times. The KLM1 pancreatic cancer cell line was provided by Dr. U. Rudloff (NCI, Bethesda, MD, USA); the L55 lung adenocarcinoma cell line was provided by Dr. S. Albelda (University of Pennsylvania, Philadelphia, PA, USA); the HAY mesothelioma cell line was provided by the Stehlin Foundation for Cancer Research (Houston, TX, USA); the gastric cancer MKN45 cell line was provided by Dr. Takao Yamori (Pharmaceuticals and Medical Device Agency, Tokyo, Japan).

### 4.4. In Vitro Furin Cleavage

Immunotoxins (2 µg) were diluted into furin cleavage buffer (100 mM Tris-HCl pH 7.5, 1 mM CaCl_2_) and incubated with 1.5 μg of recombinant human furin (Peprotech, Rocky Hill, NJ, USA) in a total volume of 20 µL for 16 h at 37 °C. Control samples were incubated without furin. All of the samples (±furin) were then split into two and analyzed under reducing (sample reducing reagent added, Life Technologies) and non-reducing conditions on 4%–12% Bis-Tris gradient polyacrylamide gels according to the manufacturer’s instructions (Novex, Life Technologies, Waltham, MA, USA).

### 4.5. Immunotoxin Stability in Human Serum

The parental PE24 containing constructs (SS1-scFv-PE24 and SS1-Fab-PE24) and the DS3-PE24 mutants (SS1-scFv-DS3-PE24 and SS1-Fab-DS3-PE24) were diluted to 15 µg/mL into non-heat inactivated 100% human AB serum (Gemini Bio-Products, West Sacramento, CA, USA). The immunotoxins were then incubated at 37 °C for 0, 1, 6, 24 and 48 h. After incubation, the samples were diluted 1:5 in PBS and used directly in the cell growth inhibition WST-8 assay described above. The assay was repeated three times.

### 4.6. In Vivo Half-Life

Balb/c mice (6–8 weeks, averaging 20 g) were injected intravenously with 30 µg of either SS1-Fab-PE24 or SS1-Fab-DS3-PE24 (*n* = 3). Blood samples were drawn at 3, 10, 20, 40, 60, 120, and 240 min. The concentration of immunotoxins in the sera was measured in ELISA using a standard curve for each immunotoxin as described previously [34]. Briefly, microtiter plates were coated with 50 μL of MSLN-rFc (a fusion protein consisting of rabbit IgG Fc fused to the mesothelin protein) (1 μg/mL) in PBS at 4 °C overnight. Diluted standards or serum samples were applied, followed by incubation with HRP-conjugated anti-PE antibody, IP12 [35]. After washing, plates were developed using tetramethylbenzidine substrate kit (Thermo Scientific, Rockford, IL, USA). Serum half-life was calculated using a “two-phase decay” model in Graphpad Prism vs. 6.01. The animal protocol (LMB-005) was approved by the National Cancer Institute Animal Care and Use Committee and the animals handled according to institute guidelines.

### 4.7. Anti-Tumor Activity

Athymic nude mice were inoculated subcutaneously with 5 × 10^6^ KLM1 cells in Matrigel on day 0. On day 8, when the average tumor volume was 150 mm^3^, mice were randomized into three groups and treated intravenously in the tail vein with either 200 μL vehicle (0.2% HSA in PBS) (*n* = 7), 1.5 mg/kg SS1-Fab-DS3-PE24 in 0.2% HSA (*n* = 10) or 1.5 mg/kg SS1-Fab-PE24 in 0.2% HSA (*n* = 10). Mice were treated qodx3 on days 8, 10 and 12. Weights and general health were monitored during treatment. Tumors were measured every 2–3 d until volumes reached 600 mm^3^. The animal protocol (LMB-014) was approved by the National Cancer Institute Animal Care and Use Committee and the animals were handled according to institute guidelines.

### 4.8. Graphs and Statistics

Graphs were plotted and analyzed using Graphpad Prism vs. 6.01 (GraphPad Software, Inc., La Jolla, CA, USA).

## Figures and Tables

**Table 1:** Immunotoxin production yields.

Construct	Yield (mg)
SS1-scFv-PE24	26
SS1-scFv-DS1-PE24	12
SS1-scFv-DS2-PE24	6.5
SS1-scFv-DS3-PE24	13
SS1-scFv-DS4-PE24	7.7
SS1-scFv-DS5-PE24	11.8
SS1-Fab-PE24	8.3
SS1-Fab-DS3-PE24	8.9

100 mg of protein was refolded and purified with the final yields listed above.

**Table 2:** Immunotoxin IC_50_ (pM) values.

Construct	KLM1	MKN45	HAY	L55
SS1-scFv-PE24	6	2	6	22
SS1-scFv-DS1-PE24	36	15	70	191
SS1-scFv-DS2-PE24	12	7	36	102
SS1-scFv-DS3-PE24	17	8	37	98
SS1-scFv-DS4-PE24	28	9	46	100
SS1-scFv-DS5-PE24	30	19	112	132
SS1-Fab-PE24	6	2	7	47
SS1-Fab-DS3-PE24	20	6	38	86

Immunotoxin IC_50_ (pM) values were determined in a WST-8 cell growth inhibition assay.

**Table 3:** Immunotoxin stability in human serum at 37 °C.

Incubation (h)	SS1-scFv-PE24	SS1-scFv-DS3-PE24	SS1-Fab-PE24	SS1-Fab-DS3-PE24
0	5 (0.5)	14 (2.7)	6 (0.7)	15 (3.0)
1	5 (0.8)	13 (2.6)	6 (2.8)	14 (0.8)
6	6 (0.4)	12 (2.5)	9 (2.8)	15 (1.0)
24	9 * (2.1)	26 * (6.6)	10 * (1.1)	22 (2.2)

Immunotoxin IC_50_ (pM) values were determined using a WST-8 cell growth inhibition assay on the KLM1 pancreatic cancer cell line after incubations in human serum at 37 °C. Numbers in parentheses and smaller font are SEM. * *p* < 0.05 using uncorrected Fisher’s LSD when comparing immunotoxin IC_50_ at time 0 and 24 h. There were no significant differences between the immunotoxins at any time point (uncorrected Fisher’s LSD).

**Table 4:** Immunotoxin serum PE levels (ng/mL).

Minutes	Experiment 1		Experiment 2	
	SS1-Fab-PE24	SS1-Fab-DS3-PE24	SS1-Fab-PE24	SS1-Fab-DS3-PE24
3	27,682	32,139	32,202	36,114
10	19,297	23,574	26,330	29,584
20	14,171	17,061	17,338	22,608
40	7009	9742	10,260	12,627
60	4813	6296	6010	8266
120	1029	1860	1541	2175
240	156	350	215	331

Mice were injected with 30 µg of immunotoxin and the serum PE (Pseudomonas exotoxin) levels (ng/ml) were monitored over time using ELISA.

**Table 5:** Serum half-life pharmacokinetic parameters.

Parameter	Experiment 1		Experiment 2	
	SS1-Fab-PE24	SS1-Fab-DS3-PE24	SS1-Fab-PE24	SS1-Fab-DS3-PE24
Dose (µg)	30	30	30	30
Bleed volume (µL)	40	40	10	10
KFast (1/min)	0.24	0.11	0.05	0.03
KSlow (1/min)	0.03	0.02	0.02	0.01
Half-life (Fast)	2.88	6.39	14.2	22.14
Half-life (Slow)	23.01	29.77	37.64	87.91
Vd (mL)	0.85	0.80	0.83	0.76
Cl (mL/min)	0.033	0.025	0.025	0.020
AUC (ng·min/mL)	0.91 × 106	1.2 × 106	1.19 × 106	1.52 × 106

The pharmacokinetic parameter values are shown. Dose: initial immunotoxin dose given to the mice. Bleed volume: the volume of blood taken at each time point for analysis. KFast and KSlow: the rate constants for the fast and slow phases of clearance. Half-life (Fast) and Half-life (Slow): the half-lives in the fast and slow phase of clearance. Vd: distribution volume. Cl: drug clearance. AUC: Area under the Curve.

## Abbreviations

The following abbreviations are used in this manuscript:

RIT	Recombinant immunotoxins
PE	*Pseudomonas* exotoxin
PC	Pro-protein convertase
TGN	Trans-golgi network
eEF2	Elongation factor 2
ADA	Anti-drug antibodies
FCS	Furin Cleavage Site
*E. coli*	*Escherichia coli*
PBS	Phosphate buffered saline
